# Cation and Zn Accumulation in Brown Seeds of the Euhalophyte *Suaeda salsa* Improves Germination Under Saline Conditions

**DOI:** 10.3389/fpls.2020.602427

**Published:** 2020-12-14

**Authors:** Jianrong Guo, Lili Liu, Ming Du, Huaying Tian, Baoshan Wang

**Affiliations:** ^1^Shandong Provincial Key Laboratory of Plant Stress, College of Life Science, Shandong Normal University, Ji'nan, China; ^2^College of Forestry Engineering, Shandong Agriculture and Engineering University, Ji'nan, China

**Keywords:** cation content, dimorphic seed, NaCl treatment, salinity tolerance, *Suaeda salsa* L.

## Abstract

Salinity inhibits plant growth due to salt ion accumulation in plant cells and reduced absorption of other nutrients such as metal ions; however halophyte plants have evolved mechanisms to survive and thrive in high-salt conditions. The euhalophyte *Suaeda salsa* generates dimorphic seeds (black and brown), which show marked differences in germination and seedling growth under high-salt conditions. However, it is unclear whether their ionic status differs. Here, to provide insight on the role of ions in salt tolerance, we used inductively coupled plasma mass spectrometry to measure the ion contents in the dimorphic seeds from *S. salsa* plants treated with or without NaCl. We measured the macroelements Na, K, Mg, and Ca, and the microelements Mn, Fe, Zn, Cu, and Mo. NaCl-treated *S. salsa* plants produced seeds with significantly reduced metallic element contents and significantly increased Na^+^ contents. The brown seeds of *S. salsa* plants treated with 0 and 200 mM NaCl had much higher contents of K^+^, Ca^2+^, and Fe^2+^ compared with the black seeds. However, the *S. salsa* seeds (both black and brown) from NaCl-treated plants were significantly larger, and had higher germination rate and higher seedling salt tolerance compared with seeds from plants not treated with NaCl. Interestingly, we measured significantly higher Zn^2+^ contents in the brown seeds from plants treated with NaCl compared with the black seeds. This suggests that the high contents of Zn^2+^ and other cations affected seed development and salt tolerance during germination under high-salt conditions. These observations provide insight into the mechanisms of salt tolerance in this halophyte and inform efforts to increase salt tolerance in salt-sensitive species.

## Introduction

Excess salt has emerged as one of the main causes decreasing crop yields in saline, arid, and semi-arid areas. It causes injury to plants due to osmotic stress, ion toxicity, and nutrient deficits (Munns and Tester, 2008). High salinity can disrupt the absorption of mineral elements by plants, resulting in nutrient imbalance, inhibited growth and development, and redistribution of biomass (Hu et al., 2007). Plants have evolved various strategies to reduce the damage caused by saline environments, one of which is to accumulate large quantities of inorganic ions in the vacuole for osmotic adjustment and avoidance of ion toxicity (Wang et al., 2004; Han et al., 2005; Qiu et al., 2007; Munns and Tester, 2008; Yang et al., 2010). Previous studies of the distribution of mineral nutrient elements in plants under salt stress mainly focused on mineral absorption and transport in roots (Ebrahimi and Bhatla, 2012). Therefore, little is known about mineral nutrient accumulation in reproductive organs under salt stress, especially in seeds.

Ionomics is the analysis of all the ions in an organism, including the metals and non-metallic elements that are the inorganic components of biological systems (Lahner et al., 2003; Salt et al., 2008; Ding et al., 2010). Ionomics can be used to examine changes in the elemental composition of organisms associated with different stages of development, different growth conditions, or different genotypes. Inductively coupled plasma mass spectrometry (ICP-MS) and other high-throughput technologies for elemental determination provide a new opportunity for investigating the relationship between ion content and plant processes, including gene function, gene networks, growth and development, and physiological processes (Lahner et al., 2003; Salt et al., 2008).

Ion content in plants is regulated by various processes, such as ion uptake, transport, and accumulation, and can be used to evaluate the physiological status of a plant. The use and redistribution of ions in plant cells is regulated by the plant's growth and development, including physiological and metabolic processes. This regulation is reflected in the changes in the ionic composition of the plant that accompany its growth and development. Thus, changes in the ion composition of plant cells reflect the specific growth and environmental state of plants.

Salinity leads to changes in the ion content of specific tissues or specific developmental stages of plants. For example, in *Arabidopsis thaliana*, plants subjected to salt stress had an altered Ca^2+^ contents when compared with control plants (Kudla et al., 2010; Ma et al., 2019; Yang et al., 2019). Additionally, the content of other ions, such as K^+^, and Mg^2+^, were decreased in plants under salinity stress conditions (Hafsi et al., 2017; Kausar and Gull, 2019). In plants, normal development, especially seed germination, is inseparable from the absorption and distribution of mineral elements.

The seed is the most important storage organ of plants and plays a central role in plant life cycles. Seeds formed under adverse environmental conditions can be of inferior quality, severely affecting the reproductive capacity and crop yield of the plants grown from them (Kranner et al., 2010; Song et al., 2016). In some cases, an individual plant produces two kinds of seeds, a phenomenon known as seed dimorphism. Dimorphic seeds usually differ in color, size, and morphology, as well as in dormancy and germination characteristics (Imbert, 2002). Seed dimorphism is prevalent in halophytes; it may be related to the spatial and temporal variability in the salinity halophytes encounter (Ungar, 1995; Song et al., 2017) and it is an important strategy for halophytes to adapt to different environments. Plants generated from seeds of different growth conditions may display differences in biomass, photosynthesis, and reproductive capacity (Talavera et al., 2010; Guo et al., 2020a). However, it is not known whether the mineral contents differ in different types of seeds or in seeds produced in different growth environments.

*Suaeda salsa* L. is an herbaceous plant widely distributed in the saline areas of northern China. This plant adapts to saline habitats by accumulating salt ions in the vacuole and regulating cellular osmotic potential (Wang et al., 2004; Han et al., 2005; Qiu et al., 2007; Qi et al., 2009; Yang et al., 2010; Mori et al., 2011), as well as by having a highly efficient antioxidant system (Pang et al., 2005, 2011; Wu et al., 2012). *S. salsa* also displays seed dimorphism; a single plant can produce black and brown seeds (Song et al., 2008; Song and Wang, 2015). Additionally, the organic storage materials and viability of *S. salsa* seeds are affected by the concentration of NaCl in the growth medium (Guo et al., 2015, 2018). Whether the growth environment also affects the mineral content of *S. salsa* seeds is unknown.

The main aim of the present study was to investigate the cation content of the black and brown seeds produced by *S. salsa* plants grown on different NaCl concentrations, and to determine the relationship between seed mineral content and germination under saline conditions. *S. salsa* plants were subjected to 0 and 200 mM NaCl, from seed sowing to seed harvesting, and their seeds were individually hand-harvested. We used ICP-MS to analyze the cation contents of the seeds, including the macroelements Na, K, Mg, and Ca, and the microelements Mn, Fe, Zn, Cu, and Mo. Understanding the relationship between seed mineral content and salt tolerance may help to identify the patterns of mineral accumulation and distribution that influence the ability of halophytes to establish populations under saline conditions.

## Materials and Methods

### Plant Materials

*Suaeda salsa* seeds were collected from the saline habitats of Shandong province and cultured as described in Guo et al. (2015), and first-, second-, and third-generation *S. salsa* seeds grown on 0 and 200 mM NaCl were obtained. The seed size of the three generations was measured using a micrometer. The third generation of *S. salsa* seeds were collected and used for ionomics analysis.

### Germination of Seeds at Different NaCl Concentrations

To investigate the seed quality and salt tolerance of the seeds from different conditions, the three generations of seeds harvested from plants grown under control (0 mM NaCl) and 200 mM NaCl were germinated at 0, 200, or 400 mM NaCl as described in our previous study (Guo et al., 2018).

### Ion Extraction and Analysis

The two types of seeds (black and brown), obtained from plants grown on 0 and 200 mM NaCl, were washed using ultrapure water (Milli-Q Reference, Millipore, USA) three times and dried for 36 h in an oven at 50°C. For each condition, a 15 mg subsample of seeds was weighed and put in a plastic tube (17 ^*^ 100 mm, 14 mL, Falcon). Next, 1 mL of HNO_3_ (68.0%) was added to each tube and the samples were digested for about 48 h. Then ultrapure water was added to each tube to a final volume of 14 mL. The elements were measured using ICP-MS (ELAN DRC-e, PerkinElmer, USA).

The relevant cation content was calculated according to the following formula:

The cation content in the sample (μmol/g dry mass)$=\;\frac{C^{*}V}{M^{*}W}$
C is the ion content measured by ICP-MS (μg/L); V is the volume of the total liquid extract (L); M is a relative atomic mass of the ion (g/mol); W is the dry sample weight (g).
Total ion content (μmol/g dry mass) = sum of various ionic contents.
Contribution of an ion to total ion content (%) = the ion content (μmol/g dry mass)/the total ion content (μmol/g dry mass).

### Instrument Operating Parameters

Inductively coupled plasma parameters: power 1100 W, cooling gas flow (Ar) 15.0 L/min, auxiliary air flow (Ar) 1.3 L/min, carrier gas flow (Ar) 0.94 L/min.

Mass spectrometer parameters: analysis room vacuum, 6 × 10^−7^ Torr, measurement parameters for the resolution (10% peak height): 0.7 amu, dwell time 50 ms, repeat three times, measuring point 1, number of cycles 20; Quality scanning, sample analysis event 28 s, sample extraction 1 mL/min.

### Ionic Analysis of *S. salsa* Leaves, Flowers, and Seeds

To determine the relationship of ion accumulation from the maternal plants (leaves and flowers) to the seeds of *S. salsa* treated with NaCl, the leaves and the corresponding flowers and seeds were collected to determine the Na^+^, K^+^ concentrations. The *S. salsa* seedlings were cultured with rinsed river sand and were irrigated with control (without NaCl) and NaCl (200 mM NaCl) twice a day (one is in the morning and the other in the late afternoon), which were same as the mother plants (seeds from control plants were treated with control, and seeds from NaCl-treated plants were treated with NaCl), and the treatment were conducted from seeds were sown till seed maturity. The preparation and determination of Na^+^, K^+^ content were same as the previous description (Guo et al., 2018).

### Statistical Analysis

The experimental data are means ± SD of three replicates. The data were analyzed by SPSS software (version 17) and based on the ANOVA (one-way) method. Different letters in the figures and tables indicate a significant difference among the mean values (*P* < 0.05) by Duncan's test.

## Results

### Characteristics of Seeds From *S. salsa* Plants Grown on 0 and 200 mM NaCl

**Figure 1:**
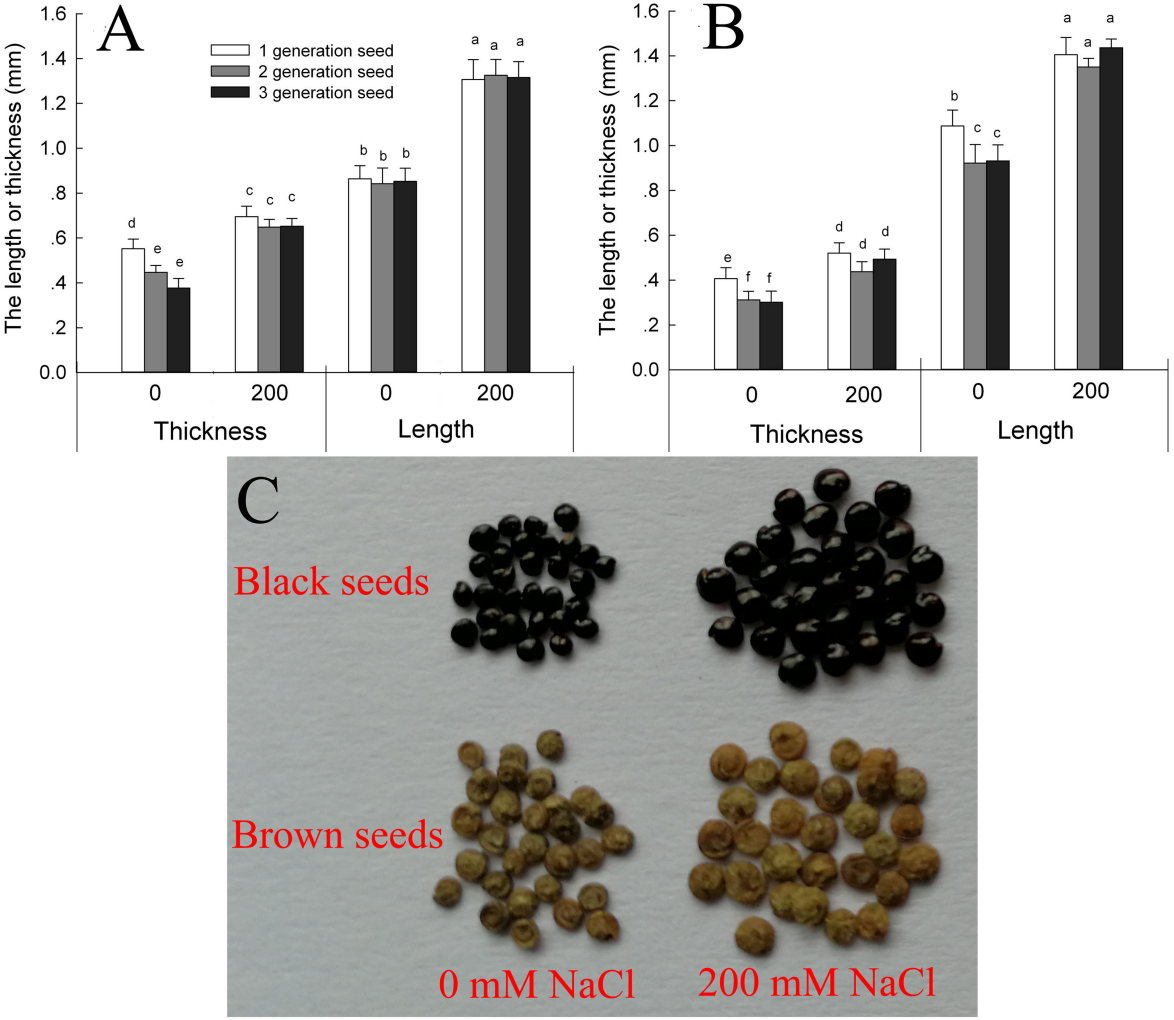
Size of black **(A)** and brown **(B)** seeds of *Suaeda salsa* from mother plants of different generations grown in 0 or 200 mM NaCl, and images of the third-generation seed **(C)**. Values are presented as means ± SD (*n* = 100). Different lower-case letters in the figure indicate significant difference at level of *P* < 0.05.

*Suaeda salsa* plants produce brown seeds and black seeds. We found that the brown seeds were significantly longer and narrower than the black seeds when the seeds were obtained from plants grown under the same conditions (Figures 1A–C). Seed development was promoted by growth on 200 mM NaCl, and seeds produced by the salt-treated plants were significantly longer and wider than those produced by control plants. For third-generation seeds, the black seeds of salt-grown plants were 152.1% longer and 125.6% wider than those of control plants, and the brown seeds were 128.7% longer and 127.9% wider. The main factor that affecting the seed size was seed source (from the maternal plant that grown with 0 or 200 mM NaCl) and seed type (black or brown seed) (Supplementary Table 1).

### Brown *S. salsa* Seeds Germinate at a Higher Rate Than Black Seeds

**Figure 2:**
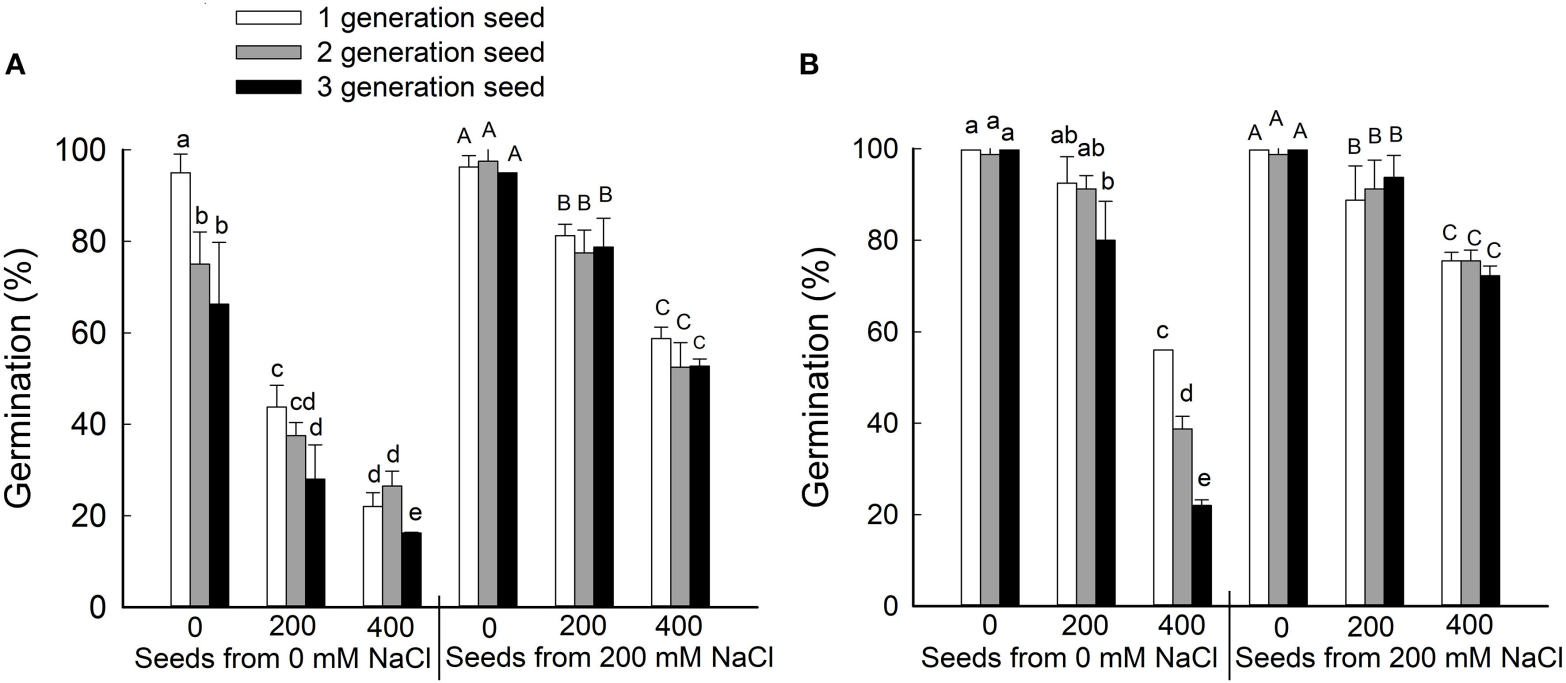
Germination percentages (%) of the black **(A)** and brown **(B)** seeds of *Suaeda salsa* from mother plants of different generations grown on 0 or 200 mM NaCl. Values are presented as means ± SD (*n* = 4). Different lower-case letters in the figure indicate significant difference at level of *P* < 0.05.

To determine whether brown and black seeds differ in their germination rate and salt tolerance, seeds were harvested from plants that had been grown for three generations on 0 or 200 mM NaCl and then germinated on 0, 200, or 400 mM NaCl (Figure 2). Generally, the germination rate decreased as NaCl concentration increased for both black and brown seeds. At each concentration of NaCl in the germination test, brown seeds had a significantly higher germination rate than black seeds, no matter whether the seeds came from 0 or 200 mM NaCl-treated parents. Seeds harvested from parent plants treated with 200 mM NaCl displayed better germination relative to seeds from control plants (Figure 2). Interestingly, we observed a significant reduction in germination rate for black seeds harvested from plants treated with 0 mM NaCl in the second and third generations, but not seeds harvested from plants treated with 200 mM NaCl (Figure 2A). No significant reduction in germination rate was observed for both seeds from NaCl-treated plants in the first, second, and third generations (Figure 2B). The main factor that affecting the seed germinate rate was seed source (from the maternal plant that grown with 0 or 200 mM NaCl) and seed type (black or brown seed) (Supplementary Table 1).

### Cation Contents of Seeds From *S. salsa* Plants Treated With 0 and 200 mM NaCl

#### Total Macroelements

Compared to the first generation, the seed size of the control plants was reduced in the second and third generations, and seeds from the third generation of the 0 and 200 mM NaCl treatments were selected for analysis of cation content. Brown seeds had more metal ions than black seeds, under both 0 and 200 mM NaCl conditions. The total ion content of the brown seeds was 1.68-fold and 1.49-fold higher than that of the black seeds harvested from plants cultured with 0 and 200 mM NaCl, respectively. Furthermore, the total cation contents of both types of seeds were lower if they came from salt-treated plants than if they came from control plants; the cation contents were 70.3 and 79.1% of control for brown seeds and black seeds, respectively.

#### Na^+^ Content

Na^+^ is the main cation present in saline environments. High concentrations of salt in the growth environment can compete with other nutrients and prevent the absorption of some essential elements, such as K and Fe. By contrast, Na is beneficial for some halophytes, helping these plants adjust their osmotic potentials when they grow in saline environments. For instance, plants in the genus *Suaeda* absorb large amounts of Na^+^ in saline environments and sequester it in vacuoles to reduce water potential and prevent ion toxicity (Flowers and Yeo, 1986).

*Suaeda salsa* seeds produced by NaCl-treated plants had significantly increased Na^+^ contents: Na^+^ was 4.6 times higher in brown seeds and 10.1 times higher in black seeds than in the corresponding seed types from control plants. In addition, in seeds from salt-treated plants, Na^+^ ions were a significantly higher percentage of the total ions (brown seeds, 32.2%; black seeds, 50.8%) than they were in seeds from control plants (brown seeds, 4.8%; black seeds, 3.9%) (Table 1). For the control plants, brown seeds accumulated 2.0 times more Na^+^ than the black seeds. By contrast, there was no significant difference in the Na^+^ contents of the two types of seeds from plants treated with 200 mM NaCl (Table 1).

**Table 1:** Macroelement contents in the black and brown seeds harvested from mother *Suaeda salsa* plants of the third generation grown on 0 or 200 mM NaCl.

**NaCl concentration (mM)**	**Black seeds**		**Brown seeds**	
	**0**	**200**	**0**	**200**
Na	72.03 ± 0.20c	724.35 ± 99.42a	147.10 ± 17.24b	685.79 ± 77.10a
K	521.71 ± 35.89b	191.57 ± 17.59d	859.14 ± 38.29a	379.31 ± 15.55c
Ca	46.23 ± 1.52b	12.25 ± 1.16d	98.91 ± 4.96a	29.30 ± 1.30c
Mg	1160.83 ± 68.05b	495.83 ± 36.94d	1919.16 ± 75.83a	1030.55 ± 70.00c
Total	1800.80	1424.01	3024.33	2124.97

Cation content, μmoles/g dry mass. Values are presented as means ± SD (n = 3).

*Different lower-case letters in the same row indicate significant difference between the seed types at level of P < 0.05*.

#### K^+^ Content

K^+^ is necessary for plant growth and development and is an important ion for osmotic adjustment. Under salt stress, plants can increase their salt tolerance by maintaining the cytoplasmic K^+^ concentration above the minimum value that is necessary to maintain normal growth under stress. For both 0 and 200 mM NaCl treatments, the K^+^ content of the black seeds was markedly lower than that of the brown seeds: the black seeds had 60.7 and 50.5% the K^+^ of brown seeds at 0 and 200 mM NaCl, respectively. The K^+^ content in both types of seeds was significantly reduced when the parent plants were treated with 200 mM NaCl: 44.1% of control for brown seeds and 36.7% of control for black seeds (Table 1). High Na^+^ competitively inhibits K^+^ uptake, which may be one reason why seeds from NaCl-treated plants had lower K^+^ contents than seeds from control plants.

#### Ca^2+^ Content

Ca^2+^ plays a vital role in growth and salt resistance in plants. As an effective membrane protectant, Ca^2+^ improves the stability of cell walls, cell membranes, and membrane-bound proteins. In the present study, NaCl treatment of *S. salsa* plants decreased the Ca^2+^ content of their seeds, by 70.4% for brown seeds and 73.5% for black seeds relative to the corresponding seed types from control plants. Brown seeds accumulated more Ca^2+^ than black seeds, regardless of the growth conditions: 1.13-fold more in control plants and 1.39-fold more in 200 mM NaCl-treated plants.

#### Mg^2+^ Content

As a component of chlorophyll and an enzyme activator, Mg^2+^ plays an important role in plant development and salt tolerance. For example, Mg^2+^ participates in lipid and phosphorus metabolism. In the present study, more Mg^2+^ accumulated in the brown seeds than in the black seeds from both salt-treated and control plants. Seeds from plants treated with 200 mM NaCl had significantly less Mg^2+^ than the control seeds: 46.3% less in brown seeds and 57.3% less in black seeds (Table 1).

### Total Microelements

Compared with the seeds harvested from control (0 mM NaCl) *S. salsa* plants, seeds harvested from treated (200 mM NaCl) plants had a reduced total microelement content in both the brown seeds (73.4% of control) and the black seeds (49.9% of control) (Table 2). Under both growth conditions, the brown seeds accumulated more microelements than the black seeds, especially Zn^2+^. Total microelement content in the brown seeds was 1.31- and 1.93-fold that of the black seeds from control and treated plants, respectively.

**Table 2:** Microelement contents in the black and brown seeds harvested from mother *Suaeda salsa* plants of the third generation grown on 0 or 200 mM NaCl.

**NaCl concentration (mM)**	**Black seeds**		**Brown seeds**	
	**0**	**200**	**0**	**200**
Fe	5035.09 ± 122.81b	2104.09 ± 106.43c	7957.89 ± 409.36a	5198.83 ± 530.99b
Mn	2927.27 ± 127.27a	1624.85 ± 123.03c	2418.18 ± 42.42b	1849.69 ± 186.67c
Cu	130.44 ± 4.52b	72.07 ± 5.55c	183.70 ± 10.37a	138.66 ± 13.93b
Zn	859.79 ± 48.08c	661.82 ± 74.95d	1181.51 ± 21.92b	1429.69 ± 83.43a
Mo	5.52 ± 0.22c	7.69 ± 0.92b	9.54 ± 0.36a	10.19 ± 0.095a
Total	8958.13	4470.52	11750.84	8627.08

Cation content, nmoles/g dry mass. Values are presented as means ± SD (n = 3).

*Different lower-case letters in the same row indicate significant difference between the seed types at level of P < 0.05*.

#### Fe^2+^ Content

As one of the essential microelements for plant growth, Fe^2+^ participates in a variety of important metabolic activities in plants. To ensure the normal growth and development of plant, a certain amount of Fe^2+^ in cells should be maintained. Fe^2+^ content in black seeds was significantly lower than in brown seeds; black seeds had 63.3 and 40.5% of the amount of Fe^2+^ in brown seeds for control and 200 mM NaCl-treated plants, respectively. Furthermore, the Fe^2+^ content was reduced by NaCl treatment, both in brown seeds (34.7% decrease) and black seeds (58.2% decrease), relative to seeds from control plants (Table 2).

#### Mn^2+^ Content

Manganese is an essential plant micronutrient involved in photosynthesis, nitrogen transformations, redox reactions, and many other enzyme activities, as well as promoting chlorophyll biosynthesis and carbohydrate transport. NaCl treatment significantly reduced the Mn^2+^ content in both types of seeds in *S. salsa*. Although there was no significant difference in Mn^2+^ content between the two types of seeds from the treated plants, black seeds accumulated 1.21-times more Mn^2+^ than brown seeds from the control plants (Table 2).

#### Cu^2+^ Content

The trends for Cu^2+^ accumulation were similar to those for Mg^2+^. For both control and treated plants, the brown seeds accumulated more Cu^2+^ than the black ones. Compared to the control, NaCl treatment led to significantly reduced Cu^2+^ content in both types of seeds (Table 2).

#### Zn^2+^ Content

Microelement deficiency could be considered to be one of the main symptoms of salt poisoning in plants. For the halophyte *S. salsa*, brown seeds had more Zn^2+^ than black seeds, regardless of the growth conditions (0 mM vs. 200 mM NaCl) of the parent plants. Unexpectedly, the Zn^2+^ content of brown seeds from plants cultured in 200 mM NaCl was significantly higher (1.21 times) than that of brown seeds from plants cultured in 0 mM NaCl. The higher Zn^2+^ content in the brown seeds might be associated with their higher salt tolerance (Table 2).

#### Mo^2+^ Content

For both control and salt-treated plants, the brown seeds had a significantly higher Mo^2+^ content than the black seeds. Surprisingly, NaCl treatment significantly enhanced the Mo^2+^ content in black seeds to 1.39 times the amount in the control black seeds. For the brown seeds there was no significant difference in Mo^2+^ content between the control and 200 mM NaCl treatment.

### Ionic Analysis of *S. salsa* Leaves, Flowers, and Seeds

To investigate the causes of the differences in ion content between the *S. salsa* seeds grown under different concentrations of NaCl, we analyzed the Na^+^ and K^+^ contents of leaves, flowers, and seeds of control and NaCl-treated plants.

#### Na^+^ Contents of *S. salsa* Leaves, Flowers, and Seeds

**Figure 3:**
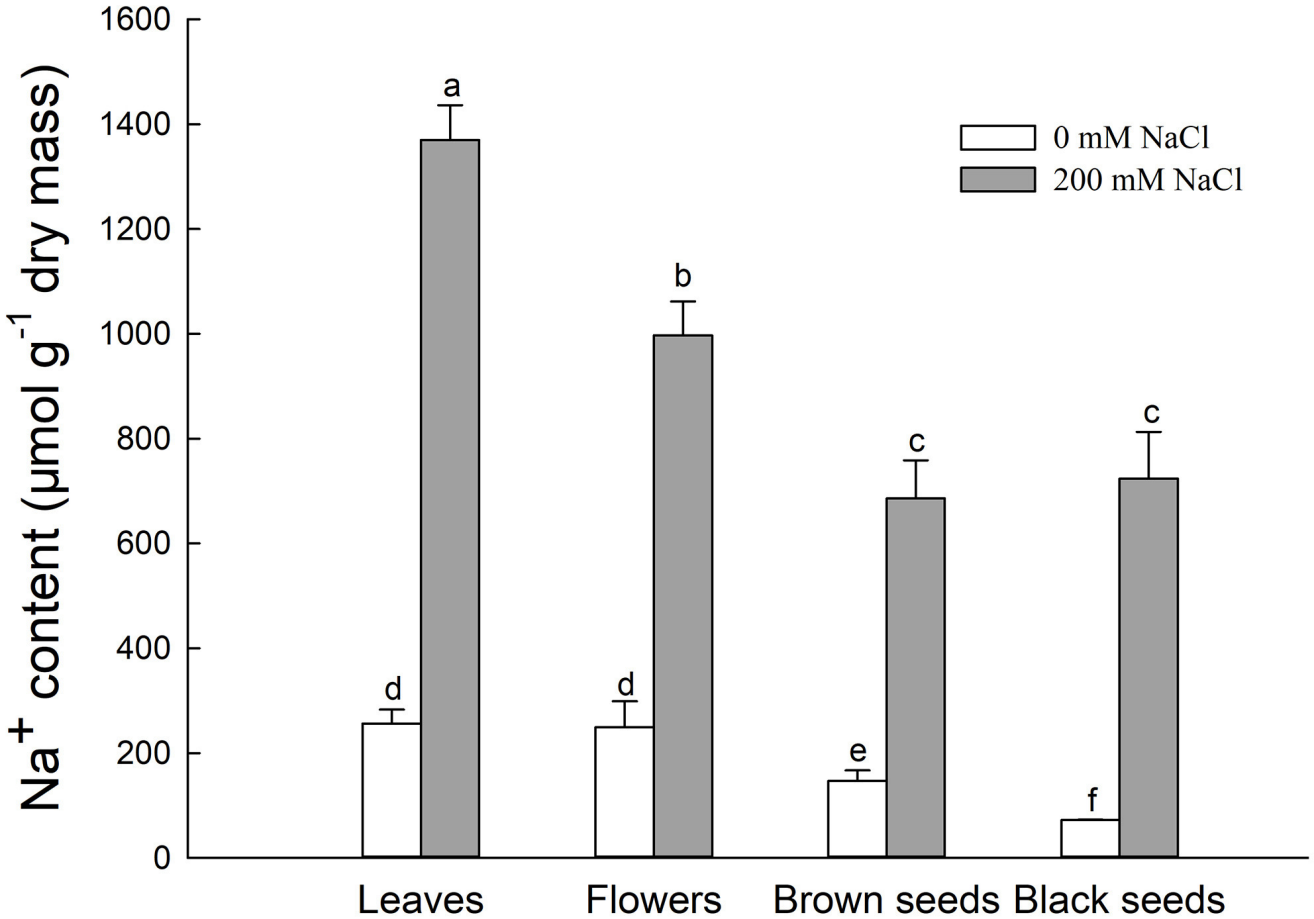
Na^+^ content in leaves, flowers, and seeds of second-generation mother *Suaeda salsa* plants grown on 0 or 200 mM NaCl. Values are presented as means ± SD (*n* = 4). Different lower-case letters in the figure indicate significant difference at level of *P* < 0.05.

The Na^+^ contents of leaves, flowers, brown seeds, and black seeds were significantly increased under the 200 mM NaCl condition, to 5.3, 5.2, 4.7, and 10.1 times the Na^+^ contents of the control, respectively (Figure 3). For NaCl-treated plants, the leaves had the highest Na^+^ content, followed by the flowers. The Na^+^ contents of the seeds were significantly lower than that of the flowers; the amounts in brown and black seeds were 68.7 and 72.5% the amount in the flowers, respectively. Thus, the Na^+^ content decreased gradually from leaf to flower to seed in the salt-treated plants. For the control plants, there was no significant difference in Na^+^ content between the leaves and the flowers, and the Na^+^ content was significantly lower in the seeds; the Na^+^ contents of brown seeds and black seeds were 58.8 and 28.8% that of the flowers, respectively (Figure 3).

#### K^+^ Contents of *S. salsa* Leaves, Flowers, and Seeds

**Figure 4:**
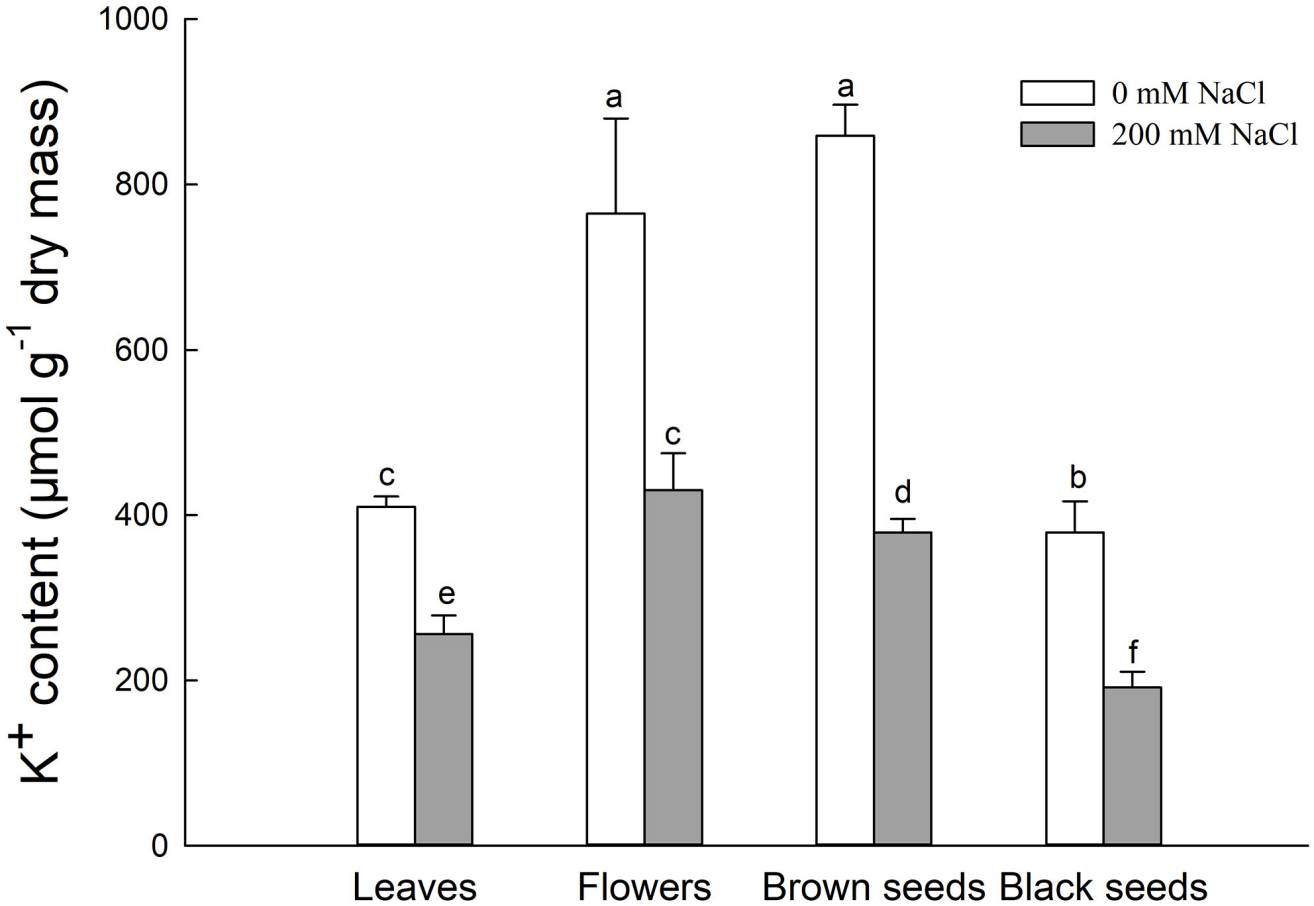
K^+^ content in leaves, flowers, and seeds of second-generation mother *Suaeda salsa* plants grown on 0 or 200 mM NaCl. Values are presented as means ± SD (*n* = 4). Different lower-case letters in the figure indicate significant difference at level of *P* < 0.05.

Generally, K^+^ uptake was competitively inhibited by excess Na^+^. The K^+^ contents in leaves, flowers, brown seeds, and black seeds of NaCl-treated *S. salsa* plants were significantly lower, by 37.6, 43.8, 55.8, and 63.3%, respectively, relative to control plants. Interestingly, for NaCl-treated plants, the flowers and brown seeds had significantly higher K^+^ contents than the leaves, while the K^+^ contents of the brown and black seeds were 11.8 and 55.5% lower than that of the flowers (Figure 4). In the control plants, the K^+^ contents of the flowers and brown seeds were also higher than that of the leaves. Although there was no significant difference between the flowers and the brown seeds of the control plants, the black seeds had 31.8% less K^+^ than the flowers. In both control and NaCl-treated *S. salsa* plants, the brown seeds had much more K^+^ than the black seeds.

#### The Na^+^/K^+^ Ratio in *S. salsa* Leaves, Flowers, and Seeds

**Figure 5:**
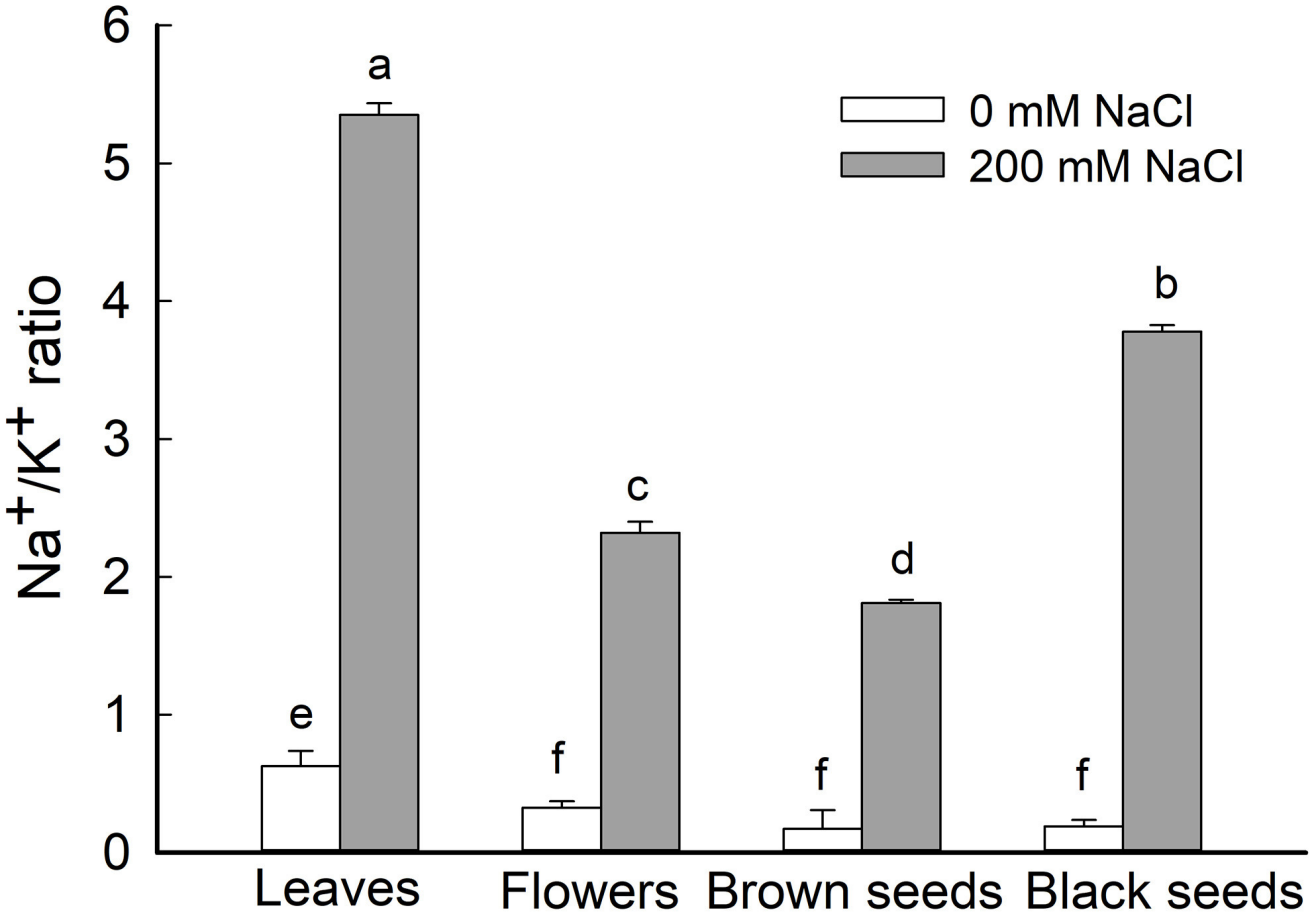
Ratio of Na^+^/K^+^ in leaves, flowers, and seeds of second-generation mother *Suaeda salsa* plants grown on 0 or 200 mM NaCl. Values are presented as means ± SD (*n* = 4). Different lower-case letters in the figure indicate significant difference at level of *P* < 0.05.

Given the general increase of Na^+^ content and decrease of K^+^ content in leaves, flowers, and seeds of NaCl-treated *S. salsa* plants, we analyzed the Na^+^/K^+^ ratio in these organs and found it to be significantly increased in the treated plants. In NaCl-treated *S. salsa* plants, the highest ratio was obtained for leaves, followed by the black seeds; the lowest ratio was observed in the brown seeds (Figure 5). For control plants, the highest Na^+^/K^+^ ratio was obtained for leaves, and this was significantly lower than the ratio for the leaves of NaCl-treated plants (Figure 5). There were no significant differences among the Na^+^/K^+^ ratios of the flowers and the brown and black seeds of the control plants.

## Discussion

In general, the presence of excess inorganic ions (mainly Na^+^ and Cl^−^) in saline soils has a toxic effect on plants, producing a hypertonic environment and inhibiting the growth of most non-halophytes (Hosseini et al., 2003; Karimi et al., 2005; Abbasi et al., 2016). As the main organ of higher plant reproduction, seeds are vital for establishing communities in specific environments, especially in ones that are unfavorable. Rapid germination and a high germination rate are important seed characteristics for halophytes to ensure normal plant growth (Tobe et al., 2004; Tlig et al., 2008), and a high salt concentration in the environment can delay seed germination (Vleeshouwers et al., 1995). Some halophytes such as *Salicornia pacifica* (Khan and Hess, 1985), *Suaeda physophora* (Song et al., 2005), and *Suaeda salsa* (Guo et al., 2018) sequester large amounts of Na^+^ ions in bracts or the pericarp, and this is important for seed germination and salt tolerance. Large amounts of K^+^ and Ca^2+^ accumulated in the hypocotyls of soybean (*Glycine max*) under high salt stress, and this protected seeds from salt stress at the pre-germination stage (Hosseini et al., 2002). Accumulation of certain ions in the seeds might directly contribute to salt tolerance during seed germination or seedling formation in saline environments. Plants have sophisticated regulatory mechanisms to maintain ionic homeostasis in seeds (Kranner and Colville, 2011).

A saline environment affects both the quality and the development of seeds (Zhou et al., 2016). In *Iris hexagona*, seeds from plants grown at an elevated salt concentration germinated rapidly and had a higher germination rate than seeds from plants grown at a lower salt concentration (Van Zandt and Mopper, 2004). Our study found that both types of seeds produced by salt-treated *S. salsa* plants were bigger than their counterparts from the control plants (Figure 1). The seeds from NaCl-treated plants also had a higher germination rate when seeds were geminated on different concentrations of salt (Figure 2). These results indicate that the presence of some NaCl in the growing medium was beneficial for seed development in the halophyte *S. salsa*, in turn contributing to improved germination and seedling emergence under saline conditions. This was especially true for the brown seeds from NaCl-treated plants, which had a higher germination rate under high NaCl conditions, even at 400 mM NaCl, despite having a higher Na^+^ content (Figure 3). The ions accumulated in seeds might directly contribute to their salt resistance, especially during germination (Song et al., 2009; Li et al., 2012), and might be beneficial to seedling establishment (Zhou et al., 2014; Guo et al., 2020a). However, the Na^+^ accumulated by the brown seeds was not the key factor improving their germination and seedling establishment because the black seeds from NaCl-treated plants had a similar Na^+^ content.

Essential macroelements are vital for plant development. In wheat, plants grown from seeds containing abundant metal ions had enhanced seedling yield and stress resistance (Marschner, 1995). The beneficial effects of metal ions in the soil are reduced by salinity, resulting in metal ion deficiencies and decreased plant quality. In this study, brown *S. salsa* seeds had significantly higher K^+^, Ca^2+^, Mg^2+^ contents than black seeds from plants treated with either 0 mM or 200 mM NaCl, although the contents of all three ions were decreased by the 200 mM NaCl treatment. Additionally, the microelements Cu^2+^, Zn^2+^, and Fe^2+^ in the brown seeds were also significantly higher than in the black seeds from plants treated with either 0 mM or 200 mM NaCl, and a decreased content was detected when treated with 200 mM NaCl. As in the seeds produced in natural habitats, the content of K^+^, Mg^2+^, and Fe^2+^ in the brown seeds was also significantly higher than that in black seeds (Zhao et al., 2018). The germination rate was also much higher for the brown seeds than for the black seeds from both NaCl-treated and control plants. The germination rate of the brown seeds from treated plants was much higher under high-salinity conditions that that of the black seeds from control (no NaCl) plants (Figure 2), indicating that maintaining high levels of K^+^, Ca^2+^, and Mg^2+^ in the seeds is very important to seed development and seed germination of the halophyte *S. salsa* under saline conditions. However, the detailed mechanism of how elevated levels of K^+^, Ca^2+^, and Mg^2+^ are maintained in brown seeds of *S. salsa* needs further study.

K^+^ is indispensable for signaling in plant growth and development, and therefore K^+^ concentrations should remain at high levels even under salinity (Wu et al., 2018; Guo et al., 2019; Wang et al., 2019). However, the K^+^ content will decrease due to high concentrations of Na^+^ in the environment and plant tissues. The K^+^ content was significantly lower in dimorphic (black and brown) seeds from treated plants compared with the controls (Table 1). These results were consistent with the K^+^ content in plants under salt stress (Hu et al., 2018). For the dimorphic seeds of NaCl-treated plants, the brown seeds had higher K^+^ contents than the black ones. This was consistent with the high salt tolerance of brown seeds from NaCl-treated plants, indicating that a high K^+^ content, rather than Na^+^, in plant cells enhanced plant growth and salt tolerance (Liu et al., 2018; Wu et al., 2018; Wang et al., 2019), which was also reflected in the Na^+^/K^+^ ratio in the flowers of treated *S. salsa* plants (Figure 5).

Zn is a microelement that is involved in salt tolerance in plants by enhancing the activity of antioxidant enzymes and protecting cell membrane integrity (Jan et al., 2017). Interestingly, the Zn^2+^ cation content was significantly lower in black *S. salsa* seeds from NaCl-treated plants, but significantly higher in the brown seeds compared with control seeds (Table 2). Additionally, the brown seeds from NaCl-treated plants showed higher salt tolerance than the black seeds (Figure 2) from NaCl-treated plants and seeds from control plants (Song et al., 2008, 2009; Guo et al., 2018). Perhaps the high salt tolerance was associated with the increased Zn^2+^ content in the brown seeds from the NaCl-treated plants. This is similar to results in *Arabidopsis thaliana*, where lines overexpressing the zinc transporter ZIP29 showed significantly higher salt tolerance than the wild type (Wang et al., 2010). Therefore, some mechanism for efficient Zn accumulation might be employed in the brown seed of *S. salsa* under salt treatment, which enhances salt tolerance and does not occur in the black seeds from NaCl-treated plants. The difference in the Zn^2+^ content between the black and brown seeds in *S. salsa* may lead to the different responses to salt in the dimorphic seeds, and thus merits further analysis.

Plants use complicated mechanisms to absorb and transport mineral elements from the soil, and to redistribute these elements in the plant, even in the seeds (Kranner and Colville, 2011). To investigate the pattern of ion accumulation in *S. salsa* plants under saline conditions, we measured the Na^+^ and K^+^ contents in leaves, flowers, and seeds. The K^+^ content in flowers and brown seeds was significantly higher than that in leaves (Figure 4), but the K^+^ content was lower than in control plants, indicating that more K^+^ was transported into the brown seeds from leaves and flowers. This K^+^ was transported along with a large amount of Na^+^ from leaves to floral organs (Figure 3), thus maintaining a relatively stable Na^+^/K^+^ ratio (Figure 5) in the flowers. The K^+^ likely enhanced the salt tolerance of brown seeds that germinated under high salt conditions (Figure 2). Therefore, *S. salsa* seems to have a mechanism for efficient transport of K^+^ from the flower to the seed, and shows a positive effect of K^+^ retention in the seed, despite the high content of Na^+^ in flowers and seeds of the treated *S. salsa* plants. The high Na^+^ content in the leaves and flowers was beneficial to form succulent leaves in *S. salsa* (Qi et al., 2009) and floral organs (mainly succulent in the petals).

Along with the K^+^, more anions (such as Cl^−^ and ${\text{NO}}_{3}^{-}$) would be absorbed to balance the cation accumulation and ensure the growth and development of plants under salinity (Song et al., 2009; Guo et al., 2018). However, the high Na^+^ and Cl^−^ contents did not inhibit the development of seeds in *S. salsa* treated with NaCl, but rather enhanced seed development; this enhancement was associated with efficient photosynthesis and photosynthetic products accumulation in the reproductive organs (Guo et al., 2020b). The enhancement was also associated with the regulation of plant hormones (Guo et al., 2020c), and the maintenance of K^+^ and Zn^2+^ content in a certain range to indirectly ensure successful reproduction of *S. salsa*.

In conclusion, the accumulation of cations such as K^+^ and Zn^2+^, but not Na^+^, in seeds from NaCl-treated *S. salsa* plants appears to be an adaptive mechanism that helps *S. salsa* survive and reproduce in saline conditions. The *S. salsa* seeds (both black and brown) from salt-treated plants were significantly larger than those from control conditions. Furthermore, the seeds had a higher seed quality and salt tolerance than seeds from plants without NaCl, despite the lower contents of K^+^, Ca^2+^, and Fe^2+^ in brown seeds. The seed development and seed quality was not affected by the reduced contents of these ions and higher Na^+^ content. Especially for brown seeds from NaCl-treated plants, the cation accumulation, particularly Zn^2+^, may enhance seed development and salt tolerance during germination. However, the full confirmation is needed to further analyze. Further research will be required to uncover the detailed mechanism of cation accumulation, particularly K^+^ and Zn^2+^, in brown *S. salsa* seeds.

## Data Availability Statement

The original contributions presented in the study are included in the article/Supplementary Material, further inquiries can be directed to the corresponding authors.

## Author Contributions

JG and BW conceived the original project, designed the experiments, and wrote the article. LL and MD performed most of the experiments. LL and HT performed the statistical analysis. All authors contributed to the article and approved the submitted version.

## Conflict of Interest

The authors declare that the research was conducted in the absence of any commercial or financial relationships that could be construed as a potential conflict of interest.
