# Supplementary material for: Cation and Zn Accumulation in Brown Seeds of the Euhalophyte Suaeda salsa Improves Germination Under Saline Conditions
Source: Front Plant Sci. 2020 Dec 14;11:602427. doi: 10.3389/fpls.2020.602427 (PMC7767863; doi:10.3389/fpls.2020.602427)
Supplement: Supplementary file 1 [file Table_1.DOCX]

**Table S1** 2-way ANOVA analysis of variance in the seed characteristics of *S. salsa* that were generated from mother plants grown in 0 or 200 mM NaCl conditions, and the treatment that same as the mother plants for three generations.

| **Dependent variable** | **Factors** | **Significant** |
| --- | --- | --- |
| Seed length | Treatment | 0.00 |
|  | Generation | 0.00 |
|  | Treatment*generation | 0.00 |
| Seed thickness | Treatment | 0.00 |
|  | Generation | 0.00 |
|  | Treatment*generation | 0.00 |
| Seed germination | Treatment | 0.00 |
|  | Generation | 0.016 |
|  | Treatment*generation | 0.00 |
